# Chronic glycemic control influences the relationship between acute perioperative dysglycemia and perioperative outcome

**DOI:** 10.1111/1753-0407.70015

**Published:** 2024-10-22

**Authors:** Brandon Stretton, Andrew E. C. Booth, Joshua Kovoor, Aashray Gupta, Ammar Zaka, Suzanne Edwards, S. George Barreto, Guy Maddern, Stephen Bacchi, Mark Boyd

**Affiliations:** ^1^ Faculty of Health and Medical Science, Adelaide Medical School University of Adelaide Adelaide South Australia Australia; ^2^ Department of Medicine Central Adelaide Local Health Network Adelaide South Australia Australia; ^3^ Ballarat Base Hospital Ballarat Central Victoria Australia; ^4^ Department of Cardiothoracic Surgery Royal North Shore Hospital St Leonards New South Wales Australia; ^5^ Gold Coast University Hospital Southport Queensland Australia; ^6^ School of Public Health The University of Adelaide Adelaide South Australia Australia; ^7^ College of Medicine and Public Health Flinders University Adelaide South Australia Australia; ^8^ Department of Medicine & Research Northern Adelaide Local Health Network Adelaide South Australia Australia

**Keywords:** glycemic control, perioperative, stress hyperglycemia ratio

## Abstract

**Background:**

The objective of this study was to evaluate the impact of dysglycemia on perioperative outcomes, in patients with and without diabetes, and how prior glycemic control modifies these relationships.

**Methods:**

Consecutive surgical patients admitted to six South Australian tertiary hospitals between 2017 and 2023 were included. Blood glucose levels within 48 h pre‐ and post‐operatively were assessed in an adjusted analyses against a priori selected covariates. Dysglycemia metrics were hyperglycemia (>10.0 mmol/L), hypoglycemia (<4.0 mmol/L), glycemic variability (standard deviation of mean blood glucose >1.7 mmol/L), and stress hyperglycemic ratio (SHR). The primary outcome was hospital mortality.

**Results:**

Of 52 145 patients, 7490 (14.4%) had recognized diabetes. Inpatient mortality was observed in 787 patients (1.5%), of which 150 (19.1%) had diabetes mellitus. Hyperglycemia was associated with increased mortality in patients with diabetes (odds ratio [OR] = 2.99, 95% CI: 1.63–5.67, *p* = 0.004) but not in non‐diabetics, who instead had an increased odds of intensive care unit (ICU) admission if hyperglycemic (OR = 1.95, 95% CI: 1.40–2.72, *p* < 0.0001). Glycemic variability was associated with increased mortality in patients with diabetes (OR = 1.46, 95% CI: 1.05–2.01, *p* < 0.05) but not in non‐diabetics. Preoperative glycemic control (HbA1c) attenuated both of these associations in a dose‐dependent fashion. Hypoglycemia was associated with increased mortality in non‐diabetics (OR = 2.14, 95% CI: 1.92–2.37, *p* < 0.001) but not in patients with diabetes.

**Conclusions,:**

In surgical patients with diabetes, prior exposure to hyperglycemia attenuates the impact of perioperative hyperglycemia and glycemic variability on inpatient mortality and ICU admission. In patients without diabetes mellitus, all absolute thresholds of dysglycemia are associated with ICU admission, unlike those with diabetes, suggesting the need to use more relative measures such as the SHR.

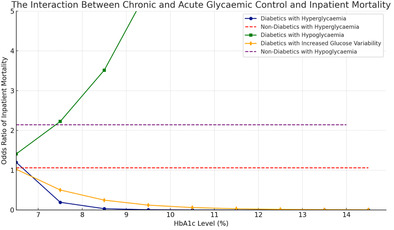

## Graphical abstract

Highlights

- Based on our study of 52 145 surgical patients, in those with diabetes, prior exposure to hyperglycemia attenuates the impact of perioperative hyperglycemia and glycemic variability on inpatient mortality and intensive care unit (ICU) admission.
- The stress hyperglycemic ratio (SHR) may represent a more accurate and pertinent predictor of perioperative outcomes than the absolute definitions by serum titer (of >10, <4 or SD >1.7). This may be because the SHR uniquely considers the antecedent glycemic control.
- In patients without diabetes mellitus, all absolute thresholds of dysglycemia are associated with ICU admission, unlike those with diabetes, suggesting the need to use more relative measures such as the SHR.

## 1 INTRODUCTION

Disordered glucose metabolism is a hallmark of the stress response to surgery and can lead to hyperglycemia, hypoglycemia, and variation in blood glucose, termed glycemic variability. These three domains of dysglycemia may occur in patients with diabetes (recognized or not), or in patients with previously normal glucose tolerance. Dysglycemia in the perioperative period is associated with adverse outcomes, including increased mortality.^1^, ^2^, ^3^, ^4^, ^5^, ^6^

Perioperative hyperglycemia, hypoglycemia, and glycemic variability exert heterogenous effects on clinical outcomes, influenced by both the nature and severity of the glucose disturbance and the patient's underlying metabolic state. These metrics do not uniformly confer the same level of risk across all patient populations. Rather, the impact of perioperative dysglycemia is context‐dependent, with diabetes status serving as a key modifier of this relationship.^1^, ^2^, ^3^, ^4^, ^5^, ^6^, ^7^, ^8^, ^9^, ^10^ In patients with diabetes, chronic dysregulation of glucose homeostasis alters the physiological response to acute fluctuations in blood glucose, potentially mitigating or exacerbating the clinical consequences of perioperative dysglycemia. Thus, the relationship between dysglycemia and adverse outcomes should be interpreted in light of both the magnitude of glucose deviation and the patient's underlying glycemic control. However, diabetes mellitus is not a binary condition, but rather there is a spectrum of glycemic control. Therefore, considering diabetic status as a binary confounder or baseline characteristic in perioperative dysglycemia analyses is not sufficiently considering the otherwise abstruse relationship between chronic and acute glycemia. This is supported by robust data from the critical care literature, where pre‐morbid glycemic control, rather than diabetes per se, attenuates the relationship between acute dysglycemia and outcome.^11^

Relationships between preoperative glycemic control, acute dysglycemia, and perioperative outcome have been infrequently reported. While earlier studies have often focused on only a single phase of the perioperative journey, such as preoperative or postoperative glycemic control, this study aimed to evaluate the entirety of the perioperative period, providing a comprehensive assessment of the impact of dysglycemia across all phases of surgery.^1^, ^6^, ^12^ This approach offers a more holistic view of the perioperative metabolic response and its implications for patient outcomes. Additionally, interpretation of this previous work is compromised by inconsistent definitions and thresholds. There are other metrics that consider a patients antecedent glucose control, such as the stress hyperglycemic ratio (SHR).^13^ Accordingly, in a large cohort of surgical patients, we aimed to evaluated the impact of preoperative glycemia on the association between metrics of dysglycemia and perioperative outcomes.

## 2 METHODS

### 2.1 Study design, setting, and population

This was an observational analysis of a prospectively maintained database. All consecutive surgical patients admitted to six South Australian tertiary adult hospitals were included. The period of study inclusion varied depending on the dates each hospital electronic medical record (EMR) implementation (2017–2023). The Central Adelaide Local Health Network Human Research Ethics Committee (reference number: 16860) provided ethics approval for this study with a waiver of consent. Patients were excluded if measurement of blood glucose level (BGL) was not performed in the perioperative period (defined as 48 h pre‐operative and 48 h post‐operative). Insulin use was determined by the prescription of any mode of insulin within 4 h of a blood glucose measurement greater than 10.0 mmol/L. Surgical procedures were defined as either elective or emergent and were further classified according to urgency (as per assigned triage codes). Surgeries were categorized as cardiac, general (breast endocrine, upper gastrointestinal, acute surgical unit, colorectal, hepato‐biliary), or “other” (a culmination of non‐cardiac, non‐general surgery).

### 2.2 Data ascertainment

Data including age, gender, patient characteristics (demographic data), biometric data and medication orders, and hospital length of stay were collected from the EMR. Surgical procedure specific data were collected from Operation Room Management Information System (ORMIS), which records surgical operations. We recorded all blood glucose measurements within 48 h either side of the surgical start time.

We identified diabetes status from the EMR and/or an HbA1c ≥6.5% within 90 days prior to the index operation.

We defined dysglycemia in “absolute” and “relative” terms. Absolute dysglycemia was defined as hyperglycemia (>10 mmol/L),^14^ hypoglycemia (<4.0 mmol/L),^8^ and increased glycemic variability (standard deviation of mean BGL >1.7 mmol/L)^6^ within 48 h pre‐ and post‐operatively.

Relative perioperative dysglycemia was defined by the SHR. SHR utilizes the estimated average blood glucose (eAG), which reflects glucose control from the previous 3 months and calculated using the equation 1.59 × *HbA1c* −2.59, where HbA1c is expressed as a percentage. SHR in turn was calculated by dividing the most proximal postoperative blood glucose by the eAG (*SHR = BGL/eAG*), representing the relative difference from eAG.^13^

The primary outcome was inpatient mortality. Secondary outcomes of interest were intensive care unit (ICU) admission, length of stay, and readmission within 30 days.

### 2.3 Statistical analysis

Baseline characteristics were evaluated using descriptive statistics. The significance level was set at *p* < 0.05. Unadjusted analyses were initially performed for predictors against outcome. Adjusted binary logistic generalized estimated equation (GEE) models were then performed for the outcomes: inpatient mortality, ICU admission, 30‐day readmission, against predictors and confounders. An adjusted linear mixed‐effects model was performed for the outcome: natural logarithm of length of stay against predictors and confounders. To accommodate for delays in clinical coding, reduce confounding, and requirement for imputation, adjusted analyses were performed on a smaller cohort with inclusion dates 2017–2022. No intentional follow‐up was conducted beyond hospital discharge, as the primary aim of the study was focused on perioperative outcomes. However, for the analysis of 30‐day readmissions, the follow‐up period was defined as 30 days from the date of discharge. Therefore, the exact follow‐up window varied depending on the patient's discharge date, and all readmissions within 30 days post‐discharge were captured. Normally distributed residuals and homoscedasticity were found to be upheld by inspection of histogram and scatter plot of residuals and predicted values only when the logarithmic transformation of the outcome: length of stay was used. Clustering on site was adjusted for as a random effect. SHR was analyzed as a continuous variable and categorical, with SHR >1.14 selected a priori as threshold of significance.^15^, ^16^ Kaplan–Meier curves and log‐rank tests were performed using R (the R foundation for statistical computing) to compare survival curves. Data visualization was performed using Python with the Matplotlib library, a widely used plotting library that offers comprehensive tools for creating static, interactive, and animated visualizations in Python. The statistical software used was SAS On Demand for Academics (SAS Institute Inc. 2021). A *p* value ≤ 0.05 was considered to be statistically significant.

## 3 RESULTS

Between January 2017 and April 2023, there were 52 145 patients who underwent surgery and had perioperative BGLs. In total, 6773 (13.0%) patients had a known diagnosis of diabetes mellitus. A further 717 patients were identified as diabetic by via an elevated preoperative HbA1c. Subcutaneous insulin in community was used by 2437 (32.5%) patients prior to their admission. Preoperative HbA1c were available on 2504 patients, with a range of 6.5%–18.5%. A total of 1 627 075 blood glucose measurements were taken across the study period (1 362 411 capillary, 264 664 venepuncture). Patients with diabetes had more frequent blood glucose monitoring in the 96‐h peri‐operative window than non‐diabetics. In diabetics, the median number of capillary BGL checks over the 96‐h period was 46 (SD = 154.94), corresponding to approximately one measurement every 2.08 h. For venous BGL checks, the median was 4 (SD = 8.34), with an interval of roughly one measurement every 24 h. In non‐diabetics, the median number of capillary BGL checks was 0 (SD = 70.45), indicating sparse or no measurements. Venous BGL checks in non‐diabetics had a median of 3 (SD = 6.52), corresponding to a measurement every 32 h.

Compared to non‐diabetics, patients with diabetes were more frail and co‐morbid, had higher BMI, were more likely to require ICU admission, and had longer hospital length of stay. Baseline characteristics are presented in Table 1.

**TABLE 1:** Baseline characteristics.

Baseline characteristic	Non diabetic (*n* = 44 655)	Diabetic (*n* = 7490)	*p* Value
Age (median [IQR])	40 (59, 75)	57 (69, 78)	**<0.0001**
Female, *N* (%)	19 795 (44.3%)	2879 (38.4%)	**<0.0001**
Hospital frailty index (median [IQR])	1.7 (3.6, 6.9)	2.1 (4.2, 7.8)	**<0.0001**
ASA (median [IQR])	2 (2, 3)	3 (3, 3)	**<0.0001**
Charlson comorbidity score (median [IQR])	2 (3, 4)	3 (5, 6)	**<0.0001**
Body mass index (median [IQR])	23.30 (26.88, 31.62)	25.00 (29.19, 34.37)	**<0.0001**
Creatinine (mmol/L) (median [IQR])	61 (75, 92)	65 (87, 127)	**<0.0001**
Emergency operation (*n* [%])	42 737 (95.7%)	6849 (91.4%)	**<0.0001**
SGLT2i use (*n* [%])	423 (0.9%)	662 (8.8%)	**<0.0001**
Inpatient intravenous insulin infusion (*n* [%])	1535 (3.4%)	2166 (28.9%)	**<0.0001**
General surgery (*n* [%])	17 747 (39.7%)	2220 (29.6%)	**<0.0001**
Cardiac surgery (*n* [%])	1697 (3.8%)	498 (6.6%)	**<0.0001**
Subspecialty surgery (*n* [%])	25 211 (56.5%)	4772 (63.7%)	**<0.0001**
Perioperative hyperglycemia (*n* [%])	7170 (16.1%)	5691 (76.0%)	**<0.0001**
Perioperative hypoglycemia (*n* [%])	2357 (5.3%)	798 (10.7%)	**<0.0001**
Perioperative increased glycemic variability (*n* [%])	4643 (10.4%)	4456 (59.5%)	**<0.0001**

Abbreviations: ASA, American Society of Anesthesiologists physical status score; SGLT2, sodium‐glucose transport protein 2 inhibitor.

In the total cohort, there were 3155 hypoglycemic episodes, 12 861 hyperglycemic episodes, and 9099 episodes of increased glycemic variability.

### 3.1 Inpatient mortality

Inpatient mortality was observed in 787 patients (1.5%), of which 150 (19.1%) had diabetes mellitus. In this diabetic cohort of non‐survivors, 126 (84%) experienced perioperative hyperglycemia, 27 (18%) experienced hypoglycemia, and 90 (60%) had increased glycemic variability. Of the 637 non‐survivors without diabetes, 243 (38.1%) experienced hyperglycemia, 89 (14.0%) experienced hypoglycemia, and 162 (25.4%) experienced increased glycemic variability. Kaplan–Meier curves of inpatient survival based on perioperative dysglycemia and SHR are provided in the supplement (Figure S1).

For patients without diabetes, in adjusted binary GEE models, only perioperative hypoglycemia demonstrated a statistically significant association with inpatient mortality (OR = 2.14, 95% CI: 1.92–2.37, *p* < 0.0001) (Table 2) Notably, perioperative hyperglycemia in the unadjusted model is significantly associated with mortality (OR = 3.30, 95% CI: 3.12–3.50, *p* < 0.0001). In adjusted analyses, the direction of relationship between dysglycemia metric and inpatient mortality did not change when differentiated by phase of surgery (Table S1). However, higher odds of inpatient mortality were observed in non‐diabetics who had hypoglycemia in the preoperative phase (OR = 2.34, 95% CI: 1.31–4.18, *p* = 0.0041) as compared to postoperative period (OR = 1.83 95% CI: 1.22–2.75, *p* = 0.0036).

**TABLE 2:** Measures of perioperative dysglycemia versus inpatient mortality.

		Diabetic cohort			Non‐diabetic		
Metric of perioperative dysglycemia		Sample size	Odds ratio (95% CI)	*p* Value	Sample size	Odds ratio (95% CI)	*p* Value
Perioperative hyperglycemia	Unadjusted	7490	**1.67 (1.33, 2.12)**	**<0.0001**	44 655	3.30 (3.12, 3.50)	<0.0001
	Adjusting for HbA1c	1521	0.76 (0.32, 1.79)	0.5277			
	Adjusted	2296	**2.99 (1.63, 5.47)**	**0.0004**	8234	1.06 (0.85, 1.32)	0.6032
	Adjusting for HbA1c	556	**0.16 (0.08, 0.31)**	**<0.001**			
Perioperative hypoglycemia	Unadjusted	7490	**1.87 (1.33, 2.62)**	**0.0003**	44 655	2.99 (2.23, 4.00)	<0.0001
	Adjusting for HbA1c	1521	1.38 (0.74, 2.58)	0.3143			
	Adjusted	2296	1.12 (0.56, 2.25)	0.7389	8234	**2.14 (1.92, 2.37)**	**<0.0001**
	Adjusting for HbA1c	556	**1.58 (1.27, 1.97)**	**<0.0001**			
Perioperative increased glucose variability	Unadjusted	7490	1.02 (0.98, 1.06)	0.2892	**44 655**	**3.01 (2.82, 3.21)**	**<0.0001**
	Adjusting for HbA1c	1521	0.91 (0.67, 1.22)	0.5112			
	Adjusted	2296	**1.46 (1.05, 2.01)**	**0.0233**	8234	0.98 (0.76, 1.26)	0.8817
	Adjusting for HbA1c	556	**0.49 (0.30, 0.78)**	**0.0030**			
Stress hyperglycemic response	Categorical >1.14 (unadjusted)	1521	1.18 (0.94, 1.48)	0.1636			
	Categorical >1.14 (adjusted)	556	1.75 (0.55, 5.56)	0.3428			
	Continuous, per 1 unit increase (unadjusted)	1521	1.05 (0.77, 1.45)	0.7412			
	Continuous, per 1 unit increase (adjusted)	556	**3.28 (1.89, 5.70)**	**<0.0001**			

*Note*: Adjusting for the confounders: Age, HFRS score, CCI score, ASA score, election (elective versus emergency and triage code), preop creatinine, BMI and clustering on site.

Abbreviations: ASA, American Society of Anesthesiologists; BMI, body mass index; CCI, Charlson Comorbidity Index; CI, confidence interval; HFRS, Hospital Frailty Risk Score.

For patients with diabetes, in an adjusted binary GEE model, there was a statistically significant association between perioperative hyperglycemia and inpatient mortality (OR = 2.99, 95% CI: 1.63–5.67, *p* < 0.0001). However, when controlling for preoperative HbA1c, the odds of inpatient mortality in those with hyperglycemia decreased by 84% for every 1 unit increase in HbA1c(OR = 0.16, 95% CI: 0.08–0.31, *p* < 0.0001). The direction of this relationship with hyperglycemia and inpatient mortality did not change when considering preroperative (OR = 1.80, 95% CI: 1.01–3.22, *p* = 0.0460) or postoperative events (OR = 2.02, 95% CI: 1.08–3.75, *p* = 0.0267) (Table S1). A similar relationship was observed with increased glucose variability, which was associated with an increased odds of inpatient mortality in patients with diabetes (OR = 1.46, 95% CI: 1.05–2.01, *p* < 0.05) which decreased by 51% for every 1 unit increase in HbA1c (OR = 0.49, 95% CI: 0.30–0.78, *p* = 0.003). Hypoglycemia in the perioperative period was not significantly associated with inpatient mortality in patients with diabetes; however, a significant interaction with preoperative HbA1c was observed where the odds of inpatient mortality increases by 58% for every 1 unit increase in HbA1c (OR = 1.58, 95% CI: 1.27–1.97, *p* < 0.0001).

**FIGURE 1:**
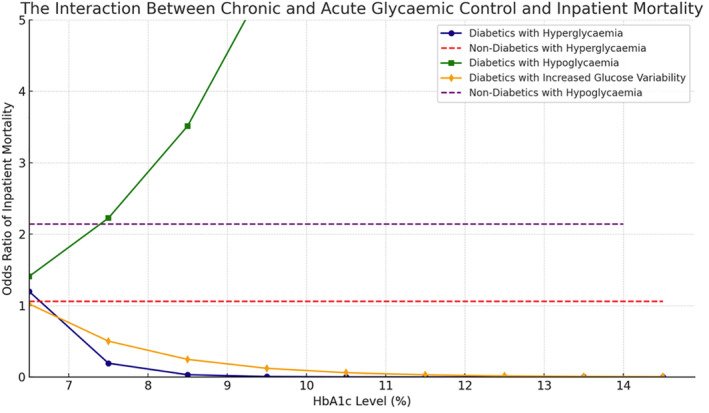
The interaction between chronic and acute glycemic control and inpatient mortality.

The interaction between acute and chronic hyperglycemia (pre‐op HbA1c) and mortality is presented in Figure 1.

When considered categorically, SHR was not associated with inpatient mortality (Table 2). However, when considered as a continuous variable, every 1 unit increase in SHR was associated with increased mortality in patients with diabetes (OR = 3.42, 95% CI: 2.20–5.31, *p* < 0.001).

### 3.2 ICU admissions

Postoperative ICU admissions were required in 8080 patients (15.5%), of which, 1265 (15.7%) had diabetes mellitus. In the diabetic cohort, 990 (78.3%) experienced perioperative hyperglycemia, 144 (11.3%) experienced hypoglycemia, and 748 (59.1%) had increased glycemic variability. Of the 6815 patients without diabetes admitted to ICU, 2149 (31.5%) experienced hyperglycemia, 457 (6.7%) experienced hypoglycemia, and 1324 (19.4%) experienced increased glycemic variability.

**FIGURE 2:**
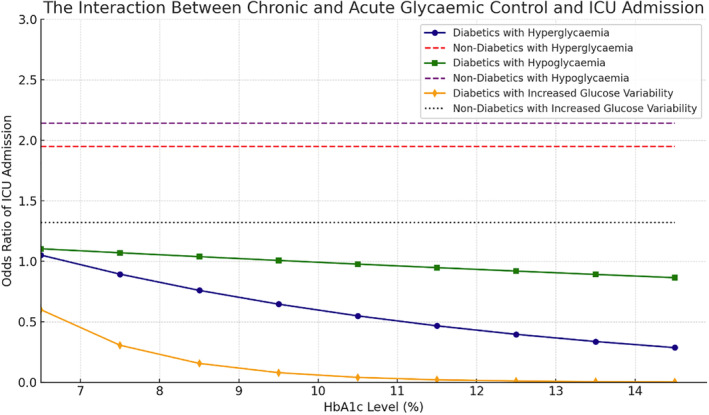
The interaction between chronic and acute glycemic control and ICU admission.

For patients without diabetes, in adjusted binary GEE models, all absolute metrics of dysglycemia were significantly associated with ICU admission (Table 3, Figure 2). Hypoglycemia conferred the most significant impact, with a 214% increased odds of ICU admission (OR = 2.14, 95% CI: 1.92–2.37, *p* < 0.0001), followed by 195% increased odds if hyperglycemic (OR = 1.95, 95% CI: 1.40–2.72, *p* < 0.0001) and 32% increased odds if there was increased glycemic variability (OR = 1.32, 95% CI: 1.19–1.47, *p* > 0.0001).

**TABLE 3:** Measures of perioperative dysglycemia versus intensive care unit admission.

		Diabetic cohort			Non‐diabetic		
Metric of perioperative dysglycemia		Sample size	Odds ratio (95% CI)	*p* value	Sample size	Odds ratio (95% CI)	*p* value
Perioperative hyperglycemia	Unadjusted	7490	1.17 (0.95, 1.43)	0.1354	44 655	**3.01 (2.35, 3.86)**	**<0.0001**
	Adjusting for HbA1c	1521	0.77 (0.57, 1.03)	0.0806			
	Adjusted	2296	1.14 (0.61, 2.12)	0.6917	8234	**1.95 (1.40, 2.72)**	**<0.0001**
	Adjusting for HbA1c	556	0.85 (0.35, 2.07)	0.7172			
Perioperative hypoglycemia	Unadjusted	7490	**1.09 (1.07, 1.12)**	**<0.0001**	44 655	1.36 (0.99, 1.86)	0.0567
	Adjusting for HbA1c	1521	0.71 (0.47, 1.09)	0.1164			
	Adjusted	2296	1.12 (0.56, 2.25)	0.7389	8234	**2.14 (1.92, 2.37)**	**<0.0001**
	Adjusting for HbA1c	556	0.97 (0.73, 1.29)	0.8354			
Perioperative increased glucose variability	Unadjusted	7490	0.98 (0.78, 1.23)	0.8744	44 655	**2.51 (2.38, 2.64)**	**<0.0001**
	Adjusting for HbA1c	1521	**0.62 (0.55, 0.69)**	**<0.0002**			
	Adjusted	2296	0.84 (0.60, 1.18)	0.3208	8234	**1.32 (1.19, 1.47)**	**<0.0001**
	Adjusting for HbA1c	556	**0.51 (0.30, 0.87)**	**0.0141**			
Stress hyperglycemic response	Categorical >1.14 (unadjusted)	1521	1.46 (1.18, 1.81)	0.0006			
	Categorical >1.14 (adjusted)	556	**1.90 (1.04, 3.48)**	**0.0372**			
	Continuous, per 1 unit increase (unadjusted)	1521	1.97 (1.68, 2.31)	<0.0001			
	Continuous, per 1 unit increase (adjusted)	556	**1.97 (1.07, 3.65)**	**0.0306**			
							

*Note*: Adjusting for the confounders: Age, HFRS score, CCI score, ASA score, election (elective versus emergency and tirage code), preop creatinine, BMI, and clustering on site.

Abbreviations: ASA, American Society of Anesthesiologists; BMI, body mass index; CCI, Charlson Comorbidity Index; CI, confidence interval; HFRS, Hospital Frailty Risk Score.

For patients with diabetes, in adjusted binary GEE models, there was no significant relationship between any absolute dysglycemia metric and ICU admission. In patients with diabetes only the SHR was associated with an increased risk of ICU admission. This association remained regardless of whether it was considered as a categorical value of >1.14 (OR = 1.90, 95% CI: 1.04–3.48, *p* = 0.0372) or continuous variable (OR = 1.97, 95% CI: 1.07–3.65, *p* = 0.0306).

## 4 DISCUSSION

A multicenter cohort study was undertaken to assess the impact of acute dysglycemia on perioperative outcomes and its interaction with chronic glycemic control. Our findings indicate that perioperative hyperglycemia, hypoglycemia and increased glycemic variability asymmetrically affects patients based on diabetic status, a relationship which is further modified by the degree of antecedent glucose control. Hyperglycemia and increased glycemic variability were associated with increased odds of inpatient mortality in patients with diabetes. In a patient with well controlled diabetes, the impact of these dysglycemia metrics is more significant than that observed in patients without diabetes. The opposite was observed in hypoglycemia, where patients with poorly controlled diabetes mellitus are conferred an increased odds of inpatient mortality compared to well controlled counterparts.

The higher odds of inpatient mortality among diabetics with hyperglycemia likely result from a selection bias which is introduced by the absolute definition thresholds. Over 75% of diabetic patients met the criteria for dysglycemia, only 15% of non‐diabetics did. Adjusting for preoperative HbA1c levels mitigates this bias. This bias also underscores a known paradox, although diabetic patients tend to be more susceptible to perioperative complications, non‐diabetic patients, when experiencing similar levels of hyperglycemia, face greater risks than their diabetic counterparts.^1^, ^5^ This is particularly pertinent with regard to ICU admission. Conversely, the association between poorly controlled diabetes and increased mortality with hypoglycemia has several potential mechanisms. Firstly, it could reflect significant metabolic dysfunction and critical illness. These patients, who typically experience chronic hyperglycemia, may have impaired counter‐regulatory mechanisms that normally protect against low BGLs. This results in a higher vulnerability to hypoglycemic episodes, which, in turn, could indicate critical illness or systemic dysfunction. This is supported by studies that have demonstrated higher rate of hypoglycemia and mortality among those with poorly controlled diabetes.^17^ Another potential mechanism is the likely concomitance of polypharmacy and comorbidities in this patient cohort, which could impair counter‐regulatory mechanisms by result of pharmacodynamic and drug–drug interactions.

In patients without diabetes, an increased odds of inpatient mortality was observed in response to hypoglycemia, but not hyperglycemia. Conversely, hyperglycemia was associated with an increased odds of ICU admission (Figure 2). With exceptions made for heterogenous definitions and outcomes, this observation is otherwise concordant with the existing literature. In their study of 7600 general surgery patients, Chen et al. defined hyperglycemia as a BGL >7.8 mmol/L, and their outcome was a composite of any National Surgical Quality Improvement Program defined postoperative complication.^5^ Patients without diabetes were conferred an increased odds of postoperative complications depending on the severity of their hyperglycemia, with adjusted odds ratios between 1.88 and 2.0. Nair et al., in their study of 2100 non‐cardiac surgery patients, those without diabetes were conferred an increased odds of ‘in‐hospital complications after surgery’ depending on the degree of hyperglycemia (OR = 1.55 to 2.26).^6^ Similar results are reported in Kotogal et al.'s study on 20 000 general surgery and spinal patients, where the odds of a “composite outcome of death, cardiac and non‐cardiac event” were increased by 60% (OR = 1.6).^1^

Our study noticeably differentiates ICU admission with inpatient mortality which may explain the difference in associations between dysglycemia and outcome. That is, many of the outcomes used in previous studies would confer the need for intensive care or high‐dependency unit, where‐in their chance of recovery is improved. The relationship between hypoglycemia and inpatient mortality, but not ICU admission, however, is potentially reflective of case specific limitations. It is likely that in patients without diabetes, the hypoglycemia is resultant of underlying illness state severity, as opposed to patients with diabetes, where this is likely medication induced, and quickly attenuated. The non‐diabetic patient who experiences hypoglycemia suggests a phenotype of low functional capacity, with limited glycemic reserves. This is the type of patient where‐in admission to ICU would be outside their ceiling of therapeutic intervention.

Our results also suggest that current guidelines for perioperative glycemic control may need adjustment. Current guidelines for the perioperative management of diabetes currently advocate for the maintenance of blood glucose between 5 and 12 mmol/L.^18^ Limited quality evidence is also referenced in preoperative HbA1c target guidelines; however, there is no evidence proving better outcomes by deferring the surgery for better glycemic control.^18^, ^19^ Our results demonstrate how poor antecedent control, reflected by high preoperative HbA1c functions to both attenuate the effect of hyperglycemia and potentially aggrandize the effect of hypoglycemia. This suggests that a more balanced, personalized approach to perioperative diabetes management is required, one that considers a patient's antecedent glycemic control. Specifically, patients with higher preoperative HbA1c's should be provided with a less restrictive glycemic target range. Such an approach is presently being advocated for in the critically ill, where patients with higher preadmission HbA1c's should be considered for more liberal glucose target ranges. Although not a pre‐specified analysis, the results, in conjunction with critical care literature, would suggest for patients with diabetes and preoperative HbA1c >8%, targets of 10–14 mmol/L could be well tolerated and reduce hypoglycemic episodes.^20^ However, such personalized perioperative targets would require further research, ideally with sequential or randomized controlled trials, before implementation.^21^, ^22^ Furthermore, patient counseling before surgery could be tailored to emphasize the importance of preoperative glycemic control, provide rationale for more liberal control in the perioperative period (if required), and encourage agency in patients engaging in their own BGL control with nutritional choices.^23^

The SHR may represent a more accurate and pertinent predictor of perioperative outcomes than the absolute definitions by serum titer (of >10, <4 or SD >1.7). This may be because the SHR uniquely considers the antecedent glycemic control. Another possibility is that a much smaller proportion of patients with a SHR >1.14 were treated with insulin than compared to those who had dysglycemia as defined by the more absolute definitions. Therefore, while SHR outperforms the other definitions in its association with perioperative outcomes, it is possible that by virtue of the barriers to calculation in an inpatient setting, clinically relevant hyperglycemia in diabetic patients is not identified or treated, conferring worse outcomes. These findings also provide a foundation for developing clinical protocols that incorporate SHR into perioperative risk stratification and lends credence to the idea of implementing artificial intelligence into clinical workflows, to aid clinicians in undertaking these higher‐level tasks in real‐time.^24^, ^25^, ^26^, ^27^ Further, when SHR was considered as a continuous variable, a significant association with mortality was found, suggesting that while the SHR threshold of >1.14 was useful in some contexts (e.g., stroke and sepsis), a more nuanced understanding of the relative increase in blood glucose may provide additional insights.^15^, ^16^ The SHR threshold of 1.14 may not be universally applicable in surgical populations, and more research is needed to determine optimal SHR targets based on patient‐specific factors and surgical risk profiles.

This study has multiple strengths. Unlike previous studies which consider diabetes mellitus as a binary covariate, or other studies which evaluate only a single phase of the patient's operative journey (pre or post); we explored the impacts of acute dysglycemia throughout the entire perioperative journey, whilst simultaneously considering diabetes as a spectrum of control.^1^, ^6^, ^12^ Further, by considering all surgical patients (cardiac and non‐cardiac), we were able to demonstrate the significance of this relationship in a broad surgical population. This study also provides novel insights into the role of the SHR as a potentially more reliable and nuanced marker of perioperative dysglycemia, offering new avenues for refining glycemic targets in surgical populations. Balanced against these strengths, there were several limitations. Capillary BGL and venous BGL are not directly comparable: Venous BGL are more accurate with respect to the laboratory testing methodology used, measured on a Roach P modular analyzer calibrated to international standards. Capillary fingerpick BGL using a glucometer is less accurate, only factory calibrated. Variability can reach up 10% more so at extremes of glucose (i.e., hypoglycemia and hyperglycemia) or during times of rapid glucose fluctuations. A majority of BGL measurements in this study were capillary. Due to the lack of standardization in frequency of BGL monitoring, the glycemic coefficient of variation was not included in this study, which may have potentially provided further insights. Whilst SGLT2 use was captured as a baseline characteristic, it was not considered in the adjusted analyses, which are a significant risk for adverse outcomes.^28^ While our study provides comprehensive insights into the relationship between perioperative dysglycemia and outcomes across a broad surgical cohort, it is important to recognize that this relationship may differ significantly in certain subgroups, such as those undergoing highly complex procedures or patients with severe comorbidities. Further research is needed to assess how these factors modify the observed associations, and whether distinct perioperative glycemic targets are necessary for different patient populations. One additional limitation is the inability to account for individual care directives or “ceiling of care” decisions. These directives typically define whether patients undergoing surgery would be eligible for ICU admission should their condition deteriorate or if they would prefer to opt for a comfort‐based, palliative approach instead. While this may affect ICU admission outcomes, it is important to note that patients undergoing surgery are generally considered candidates for ICU care unless they have (in rare situations) explicitly chosen otherwise. As such, this limitation is unlikely to have a substantial impact on our results but should still be acknowledged.

## 5 CONCLUSION

In surgical patients with diabetes, prior exposure to hyperglycemia attenuates the impact of perioperative hyperglycemia and glycemic variability on inpatient mortality and ICU admission. In patients without diabetes mellitus, all absolute thresholds of dysglycemia are associated with ICU admission, unlike those with diabetes, suggesting the need to use more relative measures such as the SHR.

## AUTHOR CONTRIBUTIONS

All authors have contributed significantly and in keeping with the latest guidelines of the International Committee of Medical Journal Editors. Conceptualzsation: Brandon Stretton, Andrew E C Booth, Joshua Kovoor, Aashray Gupta, Ammar Zaka, Suzanne Edwards, S. George Barreto, Guy Maddern, Stephen Bacchi, Mark Boyd. Methodology: Brandon Stretton, Andrew E. C. Booth, Joshua Kovoor, Suzanne Edwards, Stephen Bacchi. Validation: Brandon Stretton, Andrew E C Booth, Joshua Kovoor, Suzanne Edwards, Stephen Bacchi. Formal analysis: Brandon Stretton, Suzanne Edwards, Stephen Bacchi. Investigation: Brandon Stretton, Andrew E C Booth, Joshua Kovoor, Suzanne Edwards, Stephen Bacchi. Data curation: Brandon Stretton, Andrew E C Booth, Joshua Kovoor, Suzanne Edwards, Stephen Bacchi. Writing — original draft preparation: Brandon Stretton, Andrew E C Booth, Joshua Kovoor, Aashray Gupta, Ammar Zaka, Suzanne Edwards, S. George Barreto, Guy Maddern, Stephen Bacchi, Mark Boyd. Writing — review and editing: Brandon Stretton, Andrew E C Booth, Joshua Kovoor, Aashray Gupta, Ammar Zaka, Suzanne Edwards, S. George Barreto, Guy Maddern, Stephen Bacchi, Mark Boyd. Supervision: Guy Maddern, Stephen Bacchi, Mark Boyd.

## FUNDING INFORMATION

No direct funding to disclose.

## CONFLICT OF INTEREST STATEMENT

The authors declare no conflicts of interest.

## Supporting information


**Data S1.** Supporting Information.

## Data Availability

All data utilized is either publicly available information and appropriately referenced, or primary data that is stored under conditions granted by the CALHN HREC.

## APPENDIX A

### A.1 Length of stay

The minimum length of stay was 1 day and the maximum was 192 days. The median length of stay was 7 days (IQR = 10 days). The median length of stay for a diabetic patient was 9.2 days (IQR 10 days) and 7.25 days (IQR 8.8 days) for a patient without diabetes (*p* < 0.0001) Absolute perioperative dysglycemia was associated with increased length of stay in both diabetic and non‐diabetic patients (Table A1). In an adjusted model, perioperative hyperglycemia was associated with a mean length of stay 15% longer in patients with diabetes (Mean ratio = 1.15, 95% CI: 1.06–1.25, *p* = 0.0007) and 6% longer in patients without diabetes (Mean ratio = 1.06, 95% CI 1.02–1.10, *p* = 0.0039). Similarly, after adjustment, increased glucose variability was associated with an increased length of stay in both patients with diabetes (Mean ratio = 1.13, 95% CI 1.05–1.21, *p* = 0.0003) and without (Mean ratio = 1.06, 95% CI 1.01–1.10, *p* = 0.0165). The effects of dysglycemia on patients with diabetes was not abrogated by an elevated HbA1c (Table A1).

Perioperative hypoglycemia was only associated with an increased length of stay in patients without diabetes (Mean ratio = 1.12, 95% CI: 1.05–1.20, *p* = 0.0007).

After adjustment, the relative measure of SHR was not significantly associated with length of stay (Table A2).

**TABLE A1:** Measures of perioperative dysglycemia versus length of stay.

		Diabetic cohort			Non‐diabetic		
Metric of perioperative dysglycemia		Sample size	Odds ratio (95% CI)	*p* value	Sample size	Odds ratio (95% CI)	*p* value
Perioperative hyperglycemia	Unadjusted	7490	1.38 (1.31, 1.46)	<0.0001	44 655	1.57 (1.53, 1.61)	<0.0001
	Adjusting for HbA1c	1521	0.94 (0.84, 1.05)	0.2734			
	Adjusted	2296	**1.15 (1.06, 1.25)**	**0.0007**	8234	**1.06 (1.02, 1.10)**	**0.0039**
	Adjusting for HbA1c	556	0.90 (0.73, 1.16)	0.3501			
Perioperative hypoglycemia	Unadjusted	7490	1.17 (1.09, 1.26)	<0.0001	44 655	1.32 (1.27, 1.38)	<0.0001
	Adjusting for HbA1c	1521	0.99 (0.88, 1.12)	0.9163			
	Adjusted	2296	1.06 (0.97, 1.16)	0.1984	8234	**1.12 (1.05, 1.20)**	**0.0007**
	Adjusting for HbA1c	556	1.00 (0.86, 1.17)	0.9921	319	0.95 (0.81, 1.11)	0.5120
Perioperative increased glucose variability	Unadjusted	7490	1.32 (1.26, 1.38)	<0.0001	44 655	1.57 (1.53, 1.62)	<0.0001
	Adjusting for HbA1c	1521	0.95 (0.87, 1.03)	0.1968			
	Adjusted	2296	**1.13 (1.06, 1.21)**	**0.0003**	8234	**1.06 (1.01, 1.10)**	**0.0165**
	Adjusting for HbA1c	556	0.92 (0.79, 1.07)	0.2835	319	0.95 (0.81, 1.13)	0.5754
Stress hyperglycemic response	Categorical >1.14 (unadjusted)	1521	1.13 (1.03, 1.24)	0.0114			
	Categorical >1.14 (adjusted)	556	0.99 (0.86, 1.15)	0.9385			
	Continuous, per 1 unit increase (unadjusted)	1521	5.42 (2.18, 13.49)	0.0003			
	Continuous, per 1 unit increase (adjusted)	556	0.98 (0.84, 1.14)	0.7758			

*Note*: Adjusting for the confounders: Age, HFRS score, CCI score, ASA score, election (elective versus emergency and triage code), preop creatinine, BMI, and clustering on site.

Abbreviations: ASA, American Society of Anesthesiologists; BMI, body mass index; CCI, Charlson Comorbidity Index; CI, confidence interval; HFRS, Hospital Frailty Risk Score.

**TABLE A2:** Measures of perioperative dysglycemia versus 30‐day readmission.

		Diabetic cohort			Non‐diabetic		
Metric of perioperative dysglycemia		Sample size	Odds ratio (95% CI)	*p* value	Sample size	Odds ratio (95% CI)	*p* value
Perioperative hyperglycemia	Unadjusted	7490	0.98 (0.77, 1.24)	0.8537	44 655	**1.44 (1.33, 1.56)**	**<0.0001**
	Adjusting for HbA1c	1521	**1.03 (0.89, 1.19)**	**<0.0001**			
	Adjusted	2296	**0.76 (0.61, 0.94)**	**0.0120**	8234	1.00 (0.93, 1.06)	0.9229
	Adjusting for HbA1c	556	**1.12 (1.07, 1.16)**	**<0.0001**			
Perioperative hypoglycemia	Unadjusted	7490	**1.33 (1.16, 1.53)**	**<0.0001**	44 655	**1.21 (1.17, 1.25)**	**<0.0001**
	Adjusting for HbA1c	1521	**1.05 (0.93, 1.17)**	**0.4472**			
	Adjusted	2296	1.03 (0.73, 1.46)	0.8695	8234	1.08 (0.92, 1.26)	0.3516
	Adjusting for HbA1c	556	**1.11 (1.05, 1.17)**	**0.0002**			
Perioperative increased glucose variability	Unadjusted	7490	1.04 (0.91, 1.20)	0.5524	44 655	**1.42 (1.33, 1.53)**	**<0.0001**
	Adjusting for HbA1c	1521	**1.20 (1.00, 1.44)**	**0.0491**			
	Adjusted	2296	**0.80 (0.67, 0.94)**	**0.0073**	8234	1.00 (0.93, 1.07)	0.9602
	Adjusting for HbA1c	556	**1.14 (1.08, 1.19)**	**<0.0001**			
Stress hyperglycemic response	Categorical >1.14 (unadjusted)	1521	**1.24 (1.05, 1.47)**	**0.0118**			
	Categorical >1.14 (adjusted)	556	**1.30 (1.15, 1.48)**	**<0.0001**			
	Continuous, per 1 unit increase (unadjusted)	1521	1.14 (0.98, 1.33)	0.0846			
	Continuous, per 1 unit increase (adjusted)	556	**1.46 (1.33, 1.61)**	**<0.0001**			

*Note*: Adjusting for the confounders: Age, HFRS score, CCI score, ASA score, election (elective versus emergency and triage code), preop creatinine, BMI, and clustering on site.

Abbreviations: ASA, American Society of Anesthesiologists; BMI, body mass index; CCI, Charlson Comorbidity Index; CI, confidence interval; HFRS, Hospital Frailty Risk Score.
