# Supplementary material for: Chronic glycemic control influences the relationship between acute perioperative dysglycemia and perioperative outcome
Source: J Diabetes. 2024 Oct 22;16(10):e70015. doi: 10.1111/1753-0407.70015 (PMC11494681; doi:10.1111/1753-0407.70015)
Supplement: Supplementary file 1 — Data S1. Supporting Information. [file JDB-16-e70015-s001.docx]

SUPPLEMENTAL INFORMATION FOR PEER REVIEW

**Supplemental Information:**

Figure 1: Kaplan Meier Curves for Dysglycaemia vs Inpatient Mortality


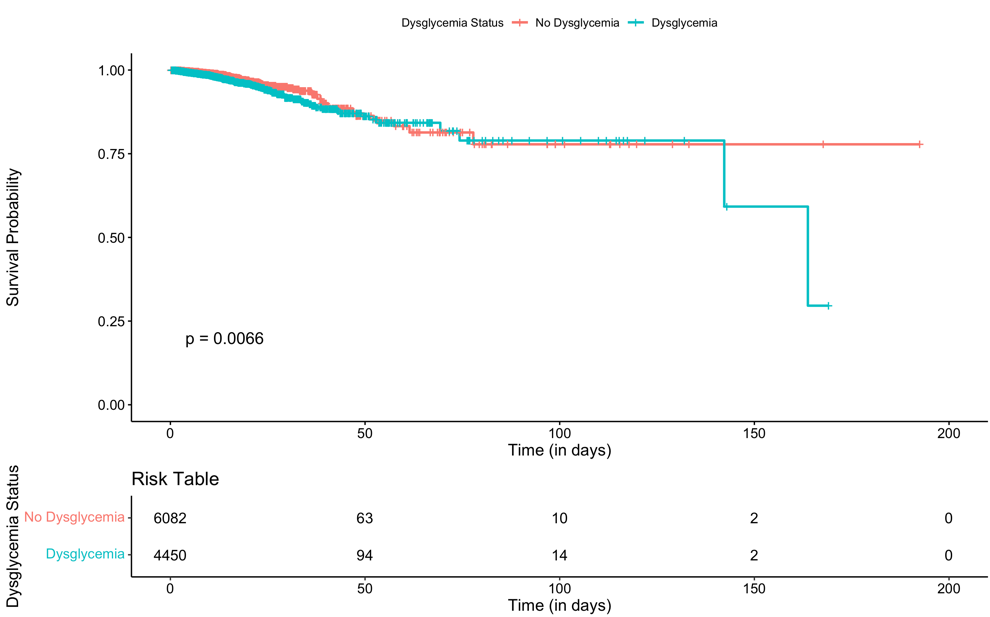
1A) Absolute Perioperative Dysglycaemia – Total Cohort

1B) Absolute Perioperative Dysglycaemia – Diabetic Cohort


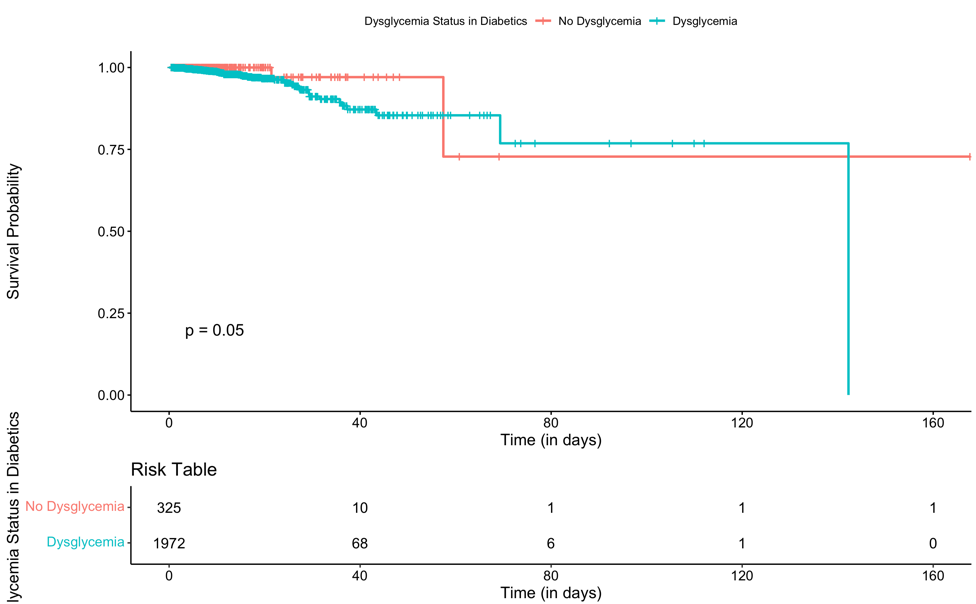


1C) Absolute Perioperative Dysglycaemia – Non-Diabetic Cohort


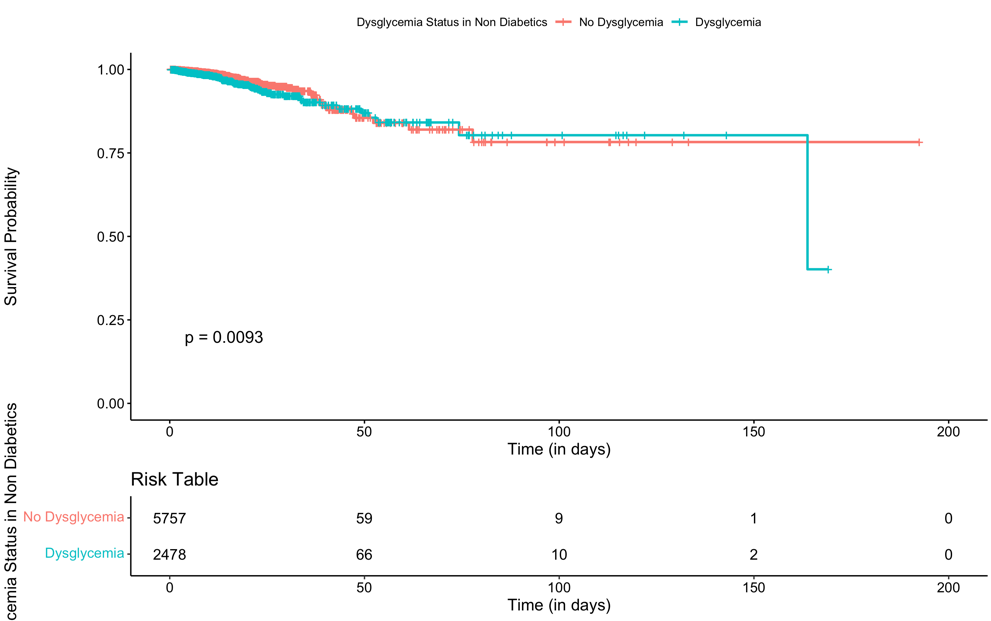


Figure 1:

1D) Stress Hyperglycaemia Response > 1.14 – Total Cohort


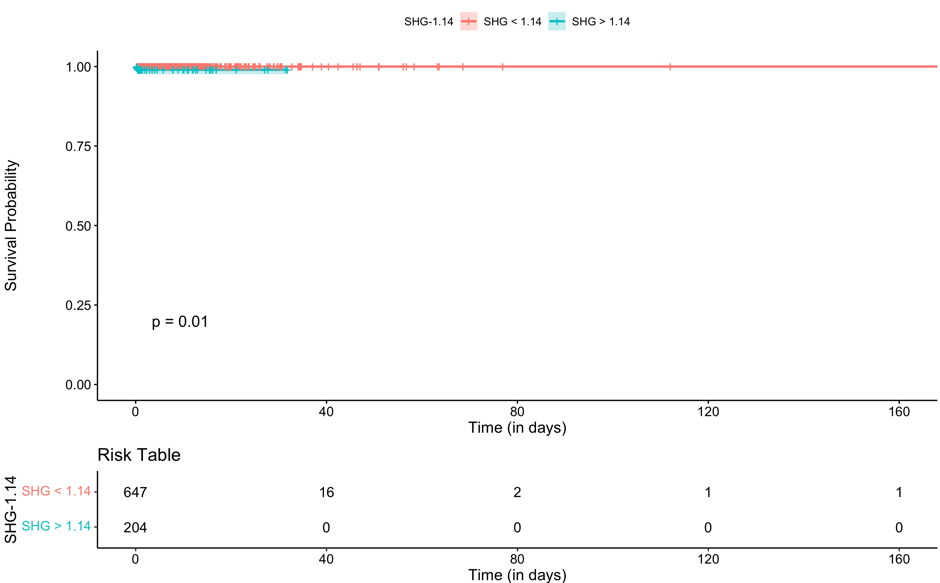


1E) Stress Hyperglycaemia Response > 1.14 – Diabetic Cohort


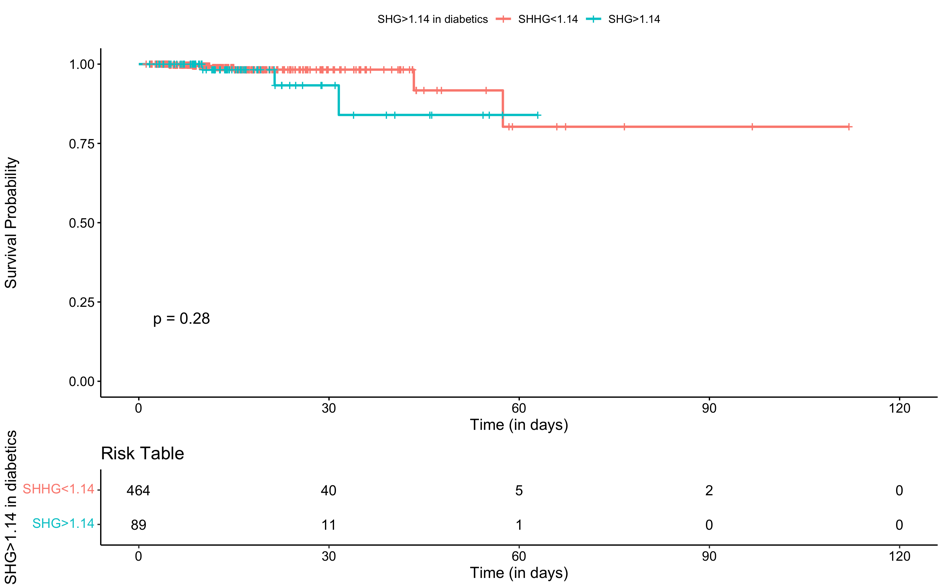


1F) Stress Hyperglycaemia Response > 1.14 – Non-Diabetic Cohort


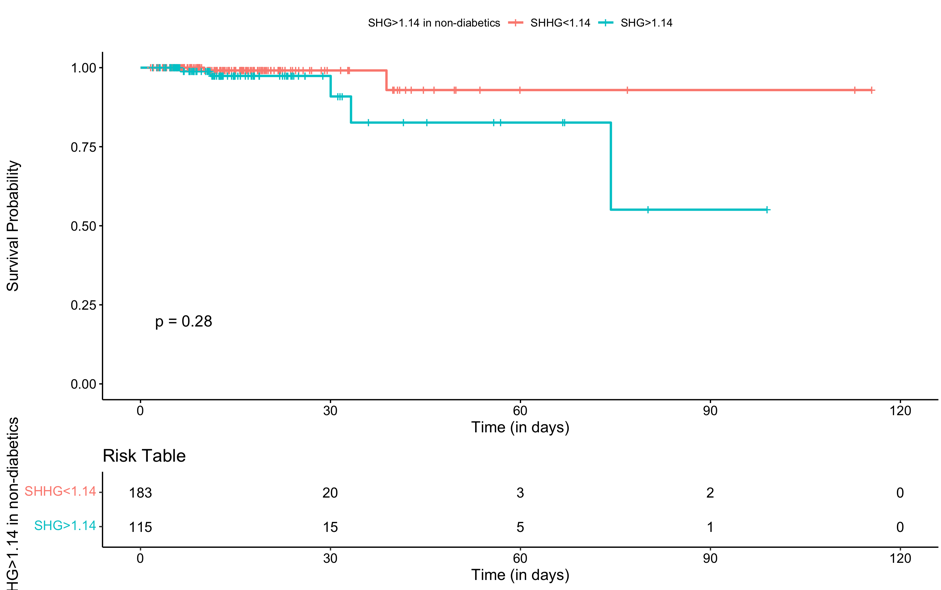


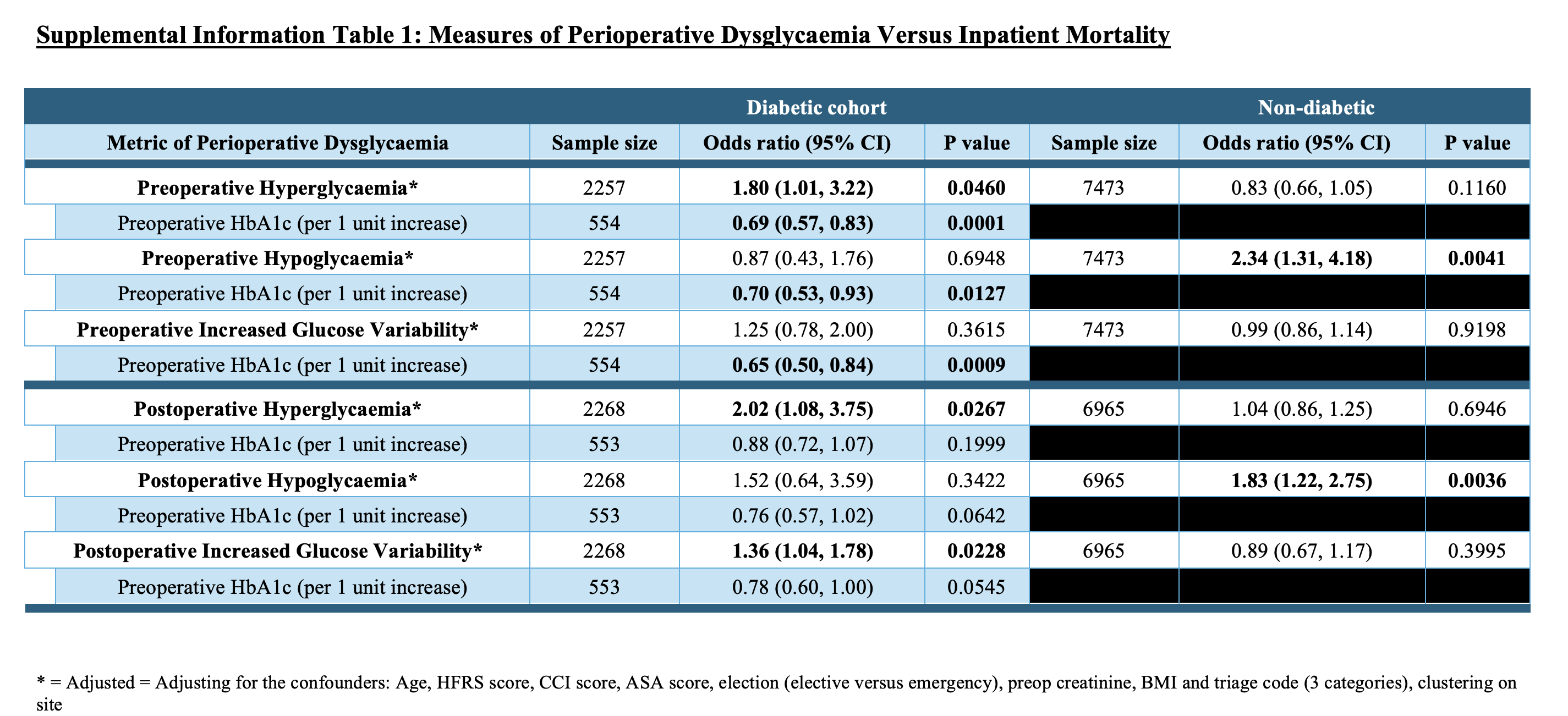


**Supplemental Table 2: Inpatient Mortality by Surgical Procedure Versus Measures of Deranged Blood Glucose Control**

| **Surgery** | **Definition of Perioperative Dysglycaemia** | **Sample Size** | **Adjustment** | **Comparison** | **Population** | **Odds Ratio (95% CI)** | **P value** |
| --- | --- | --- | --- | --- | --- | --- | --- |
| Cardiac Surgery | Absolute Perioperative Dysglycaemia | 2195 | Unadjusted | Yes vs No | Total cohort | **2.84 (2.44, 3.31)** | **<.0001** |
|  |  | 498 | Unadjusted |  | Diabetic | 0.96 (0.57, 1.60) | 0.8709 |
|  |  | 1697 | Unadjusted |  | Non-diabetic | **2.96 (2.72, 3.21)** | **<.0001** |
|  | Stress Hyperglycaemic Response | 578 | Unadjusted | Per 1 unit increase. | Total cohort | **0.38 (0.29, 0.51)** | **<.0001** |
|  |  | 241 | Unadjusted |  | Diabetic | **0.28 (0.26, 0.29)** | **<.0001** |
|  |  | 337 | Unadjusted |  | Non-diabetic | **1.66 (1.62, 1.71)** | **<.0001** |
| General Surgery | Absolute Perioperative Dysglycaemia | 19967 | Unadjusted | Yes vs No | Total cohort | **4.03 (3.17, 5.13)** | **<.0001** |
|  |  | 2220 | Unadjusted |  | Diabetic | **3.25 (2.39, 4.42)** | **<.0001** |
|  |  | 17747 | Unadjusted |  | Non-diabetic | **4.31 (3.35, 5.54)** | **<.0001** |
|  | Stress Hyperglycaemic Response | 317 | Unadjusted | Per 1 unit increase. | Total cohort | **2.43 (1.57, 3.77)** | **<.0001** |
|  |  | 198 | Unadjusted |  | Diabetic | 7.64 (0.20, 298.30) | 0.2768 |
|  |  | 119 | Unadjusted |  | Non-diabetic | 1.27 (0.52, 3.09) | 0.5935 |
| Other surgery | Absolute Perioperative Dysglycaemia | 29983 | Unadjusted | Yes vs No | Total cohort | **2.57 (2.46, 2.69)** | **<.0001** |
|  |  | 4772 | Unadjusted |  | Diabetic | 1.71 (1.13, 2.57) | 0.0105 |
|  |  | 25211 | Unadjusted |  | Non-diabetic | **3.20 (2.81, 3.66)** | **<.0001** |
|  | Stress Hyperglycaemia Response | 1608 | Unadjusted | Per 1 unit increase. | Total cohort | **2.78 (2.52, 3.07)** | **<.0001** |
|  |  | 1082 | Unadjusted |  | Diabetic | 1.16 (0.93, 1.44) | 0.1984 |
|  |  | 526 | Unadjusted |  | Non-diabetic | **4.41 (3.81, 5.12)** | **<.0001** |

Other = composite of all non-cardiac, non-general surgery procedures

**Supplemental Table 3: Intensive Care Unit Admission According to Surgical Procedure Versus Measures of Perioperative Dysglycaemia**

| **Surgery** | **Definition of Perioperative Dysglycaemia** | **Sample Size** | **Adjustment** | **Comparison** | **Population** | **Odds Ratio * (95% CI)L** | **P value** |
| --- | --- | --- | --- | --- | --- | --- | --- |
| Cardiac Surgery | Absolute Perioperative Dysglycaemia | 2195 | Unadjusted | 1 vs 0 | **Total cohort** | **3.60 (3.13, 4.15)** | **<.0001** |
|  |  | 498 | Unadjusted |  | Diabetic | 1.58 (0.78, 3.21) | 0.2014 |
|  |  | 1697 | Unadjusted |  | **Non-diabetic** | **3.96 (3.75, 4.19)** | **<.0001** |
|  | Stress Hyperglycaemic Response | 578 | Unadjusted | Per 1 unit increase. | Total cohort | 1.23 (0.62, 2.45) | 0.5551 |
|  |  | 241 | Unadjusted |  | Diabetic | 1.38 (0.14, 13.18) | 0.7813 |
|  |  | 337 | Unadjusted |  | **Non-diabetic** | **1.25 (1.13, 1.37)** | **<.0001** |
| General Surgery | Absolute Perioperative Dysglycaemia | 3508 | Adjusted | 1 vs 0 | **Total cohort** | **1.52 (1.23, 1.87)** | **<.0001** |
|  |  | 2220 | Adjusted |  | Diabetic | 1.08 (0.59, 1.95) | 0.8063 |
|  |  | 17747 | Unadjusted |  | **Non-diabetic** | **1.72 (1.26, 2.34)** | **0.0006** |
|  | Stress Hyperglycaemic Response | 317 | Unadjusted | Per 1 unit increase. | **Total cohort** | **1.59 (1.22, 2.06)** | **0.0005** |
|  |  | 198 | Unadjusted |  | Diabetic | 0.95 (0.64, 1.42) | 0.8182 |
|  |  | 119 | Unadjusted |  | **Non-diabetic** | **2.40 (1.40, 4.11)** | **0.0014** |
| Other surgery | Absolute Perioperative Dysglycaemia | 6281 | Adjusted | 1 vs 0 | Total cohort | 1.09 (0.87, 1.37) | 0.4455 |
|  |  | 4772 | Adjusted |  | Diabetic | 0.73 (0.51, 1.05) | 0.0911 |
|  |  | 25211 | Unadjusted |  | **Non-diabetic** | **1.76 (1.34, 2.31)** | **<.0001** |
|  | Stress Hyperglycaemia Response | 1608 | Adjusted | Per 1 unit increase. | **Total cohort** | **2.37 (2.07, 2.71)** | **<.0001** |
|  |  | 1082 | Unadjusted |  | **Diabetic** | **1.69 (1.25, 2.28)** | **0.0006** |
|  |  | 526 | Unadjusted |  | **Non-diabetic** | **2.31 (2.24, 2.38)** | **<.0001** |

Adjusted = Adjusting for the confounders: Age, HFRS score, CCI score, ASA score, election (elective versus emergency and triage code), preop creatinine, BMI and clustering on site

**Supplemental Table 4: Length of Stay by Surgical Procedure Versus Measures of Deranged Blood Glucose Control**

| **Surgery** | **Definition of Perioperative Dysglycaemia** | **Sample Size** | **Adjustment** | **Comparison** | **Population** | **Odds Ratio (95% CI)** | **P value** |
| --- | --- | --- | --- | --- | --- | --- | --- |
| Cardiac Surgery | Absolute Perioperative Dysglycaemia | 741 | Adjusted | 1 vs 0 | Total cohort | 1.05 (0.98, 1.12) | 0.1758 |
|  |  | 183 | Adjusted |  | Diabetic | 0.92 (0.72, 1.17) | 0.4910 |
|  |  | 558 | Adjusted |  | Non-diabetic | 1.05 (0.97, 1.13) | 0.2343 |
|  | Stress Hyperglycaemic Response | 165 | Adjusted | Per 1 unit increase. | Total cohort | 1.04 (0.86, 1.26) | 0.6706 |
|  |  | 81 | Adjusted |  | Diabetic | 0.95 (0.04, 24.32) | 0.9756 |
|  |  | 84 | Adjusted |  | Non-diabetic | 0.95 (0.07, 12.86) | 0.9714 |
| General Surgery | Absolute Perioperative Dysglycaemia | 3508 | Adjusted | 1 vs 0 | Total cohort | 1.04 (0.99, 1.09) | 0.1656 |
|  |  | 606 | Adjusted |  | Diabetic | 1.09 (0.94, 1.26) | 0.2665 |
|  |  | 2902 | Adjusted |  | Non-diabetic | 1.06 (1.00, 1.12) | 0.0631 |
|  | Stress Hyperglycaemic Response | 97 | Adjusted | Per 1 unit increase. | Total cohort | 1.23 (0.86, 1.77) | 0.2587 |
|  |  | 60 | Adjusted |  | Diabetic | 8.87 (0.13, 607.12) | 0.3038 |
|  |  | 37 | Adjusted |  | Non-diabetic | 6.39 (0.00, 21002.62) | 0.6393 |
| Other surgery | Absolute Perioperative Dysglycaemia | 6281 | Adjusted | 1 vs 0 | Total cohort | **1.12 (1.07, 1.17)** | **<.0001** |
|  |  | 1507 | Adjusted |  | Diabetic | 1.10 (0.98, 1.23) | 0.0943 |
|  |  | 4774 | Adjusted |  | Non-diabetic | **1.12 (1.06, 1.18)** | **<.0001** |
|  | Stress Hyperglycaemia Response | 613 | Adjusted | Per 1 unit increase. | Total cohort | 1.05 (0.98, 1.13) | 0.1434 |
|  |  | 415 | Adjusted |  | Diabetic | 0.83 (0.11, 6.30) | 0.8595 |
|  |  | 198 | Adjusted |  | Non-diabetic | 1.74 (0.73, 4.14) | 0.2114 |

Adjusted = Adjusting for the confounders: Age, HFRS score, CCI score, ASA score, election (elective versus emergency and triage code), preop creatinine, BMI and clustering on site

**Supplemental Table 5: 30-day Readmission According to Surgical Procedure Versus Measures of Perioperative Dysglycaemia**

| **Surgery** | **Definition of Perioperative Dysglycaemia** | **Sample Size** | **Adjustment** | **Comparison** | **Population** | **Odds Ratio (95% CI)** | **P value** |
| --- | --- | --- | --- | --- | --- | --- | --- |
| Cardiac Surgery | Absolute Perioperative Dysglycaemia | 741 | Adjusted | 1 vs 0 | Total cohort | **0.88 (0.87, 0.89)** | **<.0001** |
|  |  | 183 | Adjusted |  | Diabetic | **0.24 (0.14, 0.40)** | **<.0001** |
|  |  | 558 | Adjusted |  | Non-diabetic | 0.92 (0.80, 1.07) | 0.2818 |
|  | Stress Hyperglycaemic Response | 578 | Unadjusted | Per 1 unit increase. | Total cohort | 0.71 (0.49, 1.02) | 0.0670 |
|  |  | 241 | Unadjusted |  | Diabetic | **0.51 (0.39, 0.67)** | **<.0001** |
|  |  | 337 | Unadjusted |  | Non-diabetic | 1.06 (0.62, 1.83) | 0.8218 |
| General Surgery | Absolute Perioperative Dysglycaemia | 3508 | Adjusted | 1 vs 0 | Total cohort | **1.35 (1.17, 1.54)** | **<.0001** |
|  |  | 606 | Adjusted |  | Diabetic | 1.07 (0.72, 1.58) | 0.7327 |
|  |  | 2902 | Adjusted |  | Non-diabetic | **1.22 (1.06, 1.40)** | **0.0052** |
|  | Stress Hyperglycaemic Response | 317 | Unadjusted | Per 1 unit increase. | Total cohort | **1.77 (1.20, 2.63)** | **0.0042** |
|  |  | 198 | Unadjusted |  | Diabetic | **2.35 (1.38, 4.01)** | **0.0017** |
|  |  | 119 | Unadjusted |  | Non-diabetic | 1.42 (0.30, 6.71) | 0.6569 |
| Other surgery | Absolute Perioperative Dysglycaemia | 6281 | Adjusted | 1 vs 0 | Total cohort | **1.10 (1.04, 1.16)** | **0.0009** |
|  |  | 1507 | Adjusted |  | Diabetic | **0.74 (0.67, 0.82)** | **<.0001** |
|  |  | 4774 | Adjusted |  | Non-diabetic | 1.07 (0.98, 1.16) | 0.1547 |
|  | Stress Hyperglycaemia Response | 613 | Adjusted | Per 1 unit increase. | Total cohort | **0.83 (0.74, 0.94)** | **0.0023** |
|  |  | 415 | Adjusted |  | Diabetic | 1.13 (0.88, 1.44) | 0.3328 |
|  |  | 198 | Adjusted |  | Non-diabetic | **0.74 (0.61, 0.89)** | **0.0013** |

Adjusted = Adjusting for the confounders: Age, HFRS score, CCI score, ASA score, election (elective versus emergency and triage code), preop creatinine, BMI and clustering on site
